# Supplementary material for: A Self-Assembling Cross-Protective Antigen Against Multiple Gram-Positive Nosocomial Pathogens
Source: ACS Omega. 2025 Apr 29;10(18):19073–81. doi: 10.1021/acsomega.5c01404 (PMC12079206; doi:10.1021/acsomega.5c01404)
Supplement: Supplementary file 1 — ao5c01404_si_001.pdf [file ao5c01404_si_001.pdf]

A self-assembling cross-protective antigen against multiple Gram-positive nosocomial pathogens

Eliza Kramarska[1,4±], Felipe Romero-Saavedra[2±], Flavia Squeglia[1], Sara La Manna[3], Oceane Sadones[2], Daniela Marasco[3], Rita Berisio[1*], and Johannes Huebner[2*]

1. Institute of Biostructures and Bioimaging, Italian Research Council (CNR), Naples, Italy.
2. Division of paediatric infectious disease, Hauner children's hospital, LMU, Munich, Germany.
3. Department of Pharmacy, University of Naples Federico II, Napoli, Italy.
4. Łukasiewicz Research Network – PORT Polish Center for Technology Development, Wroclaw, Poland

[±]These authors equally contributed to this work

[*]Correspondence: rita.berisio@cnr.it; johannes.huebner@med.uni-muenchen.de;

Supplementary Material

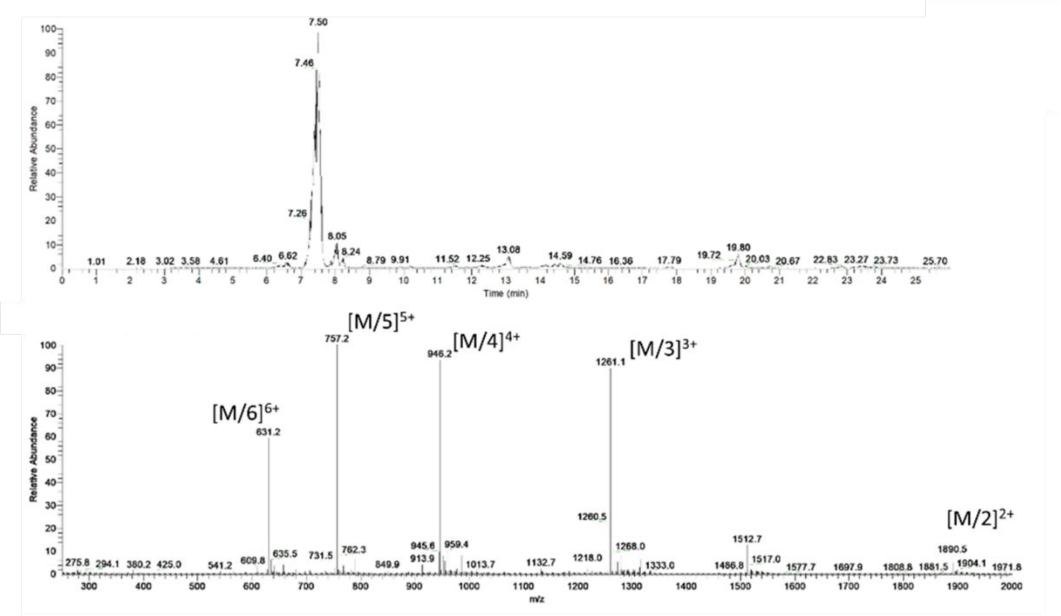

Figure S1. Upper panel: LC-MS profile, Lower panel: MS spectrum of the main peak of crude non-acetylated Q11-EH peptide.

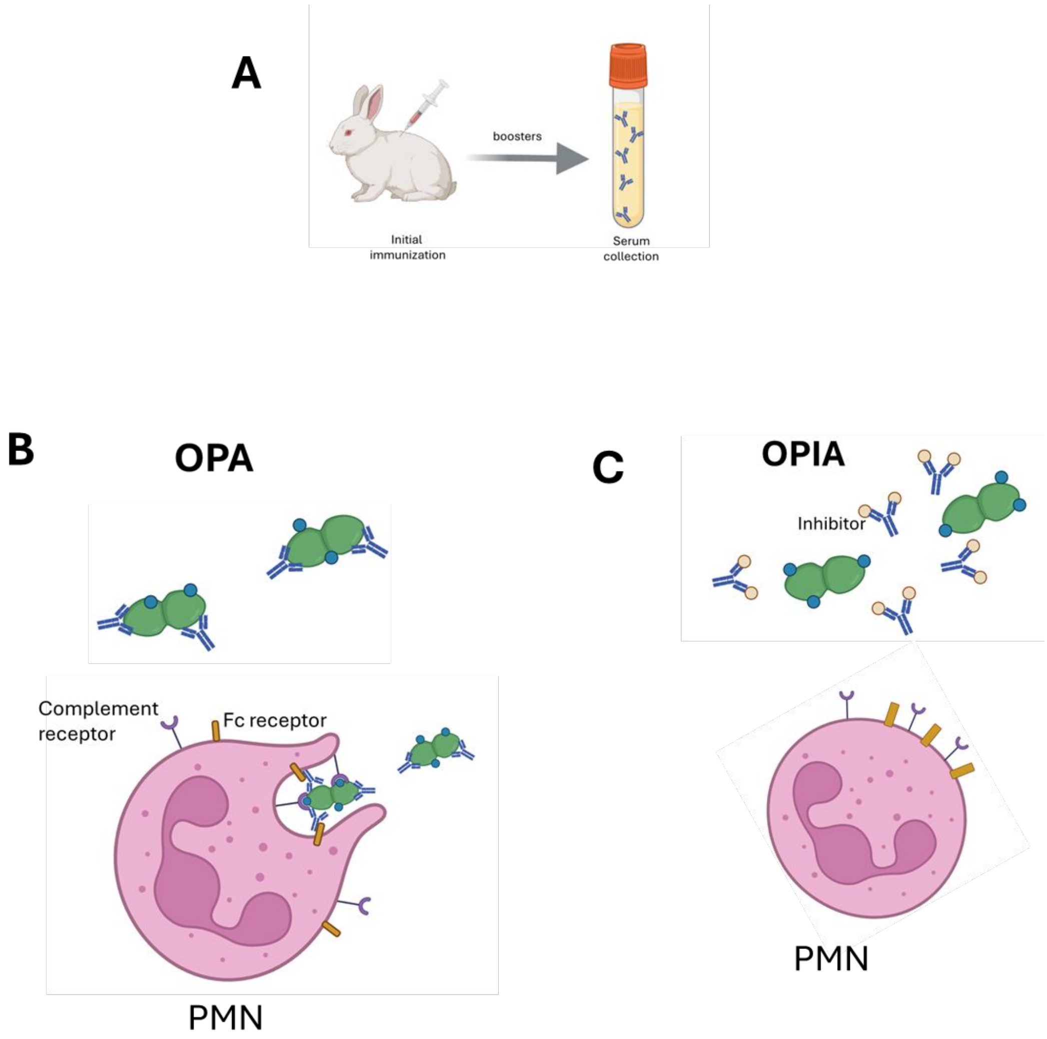

Figure S2. A schematic representation of rabbit immunization (A) and mechanism of OPA (B) and OPIA (C). In panel B, antigen-specific antibodies and complement proteins opsonise bacteria and facilitate the uptake of the antibody-bacteria complex by phagocytes (PMN). In panel C, the inhibiting antigen binds antibodies, thus hampering opsonophagocytic killing.

Table S1. Primers used for the recombinant production of AdcA and ZnuA domain

AdcA	AdcA-5-BamHI aggcGGATCCTCGAATGATAAAGATGGAAAAT<br><br>AdcA-3-PstI aggcCTGCAGTTAATGAGCCATCATTTCTTGA
ZnuA	actacGGATCCAAATTAGAAATTGTAACAAC<br><br>gactagAAGCTTAAACCATAGTTGTTTTTTCTAACGC
